# Simultaneous detection of infectious bronchitis virus and avian metapneumovirus genotypes A, B, and C by multiplex RT-qPCR assay in chicken tracheal samples in Ecuador

**DOI:** 10.3389/fvets.2024.1387172

**Published:** 2024-07-18

**Authors:** Anthony Loor-Giler, Claire Muslin, Silvana Santander-Parra, Dayana Coello, David De la Torre, Hernán Abad, Luis Nuñez

**Affiliations:** ^1^Laboratorios de Investigación, Dirección General de Investigación, Universidad de las Américas (UDLA), Quito, Ecuador; ^2^Facultad de Ingeniería y Ciencias Aplicadas, Carrera de Ingeniería en Biotecnología, Universidad de Las Américas (UDLA), Quito, Ecuador; ^3^Facultad de Ciencias de la Salud, Carrera de Medicina Veterinaria, Universidad de Las Américas, Quito, Ecuador; ^4^One Health Research Group, Universidad de Las Americas, Quito, Ecuador; ^5^LABIGEN, Laboratory of Molecular Biology and Genetics, Quito, Ecuador

**Keywords:** IBV, aMPV, multiplex, qPCR, respiratory disease, chicken

## Abstract

Respiratory RNA viruses such as Infectious bronchitis virus (IBV) and Avian metapneumovirus (aMPV), which are characterized by generating both respiratory damage and adverse effects on reproductive organs, affect poultry production economically due to high mortality rate and decrease in egg production and quality. Particularly, aMPV has three genotypes that have been reported with greater frequency in chickens: aMPV-A, aMPV-B, and aMPV-C. The present study proposes the design of a multiplex RT-qPCR assay for the simultaneous diagnosis of the 3 genotypes of interest of aMPV and IBV, followed by testing of 200 tracheal samples of vaccinated chickens with respiratory symptoms and finally a phylogenetic analysis of the sequences found. The assay detected up to 1 copy of each viral genome. The standard curves showed an efficiency between 90 and 100% in the multiplex assay and inter- and intra-assay coefficients of variation of 0.363 and 0.459, respectively and inter- and intra-assay coefficients of variation of 0.363 and 0.459, respectively. 69.5% of samples were found positive alone or in coinfection. 114 samples were positive for IBV, 13 for aMPV-A and 25 for aMPV-B. RNA of aMPV-C was no detected. The most commonly found combination was aMPV-B and IBV within 6 samples, and the least common was aMPV-A and aMPV-B in coinfection in 2 samples. The assay was specific for amplification of the genomes of the studied respiratory viruses (IBV, aMPV-A, aMPV-B, aMPV-C) as no amplification was shown from other viral genomes (ChPV, CAstV, ANV, and FAdV) or from the negative controls. Partial genomic Sanger sequencing enabled to identify circulating vaccine-derived and wild-type strains of IBV and vaccine and vaccine-derived strains of aMPV-B. In conclusion, this newly developed multiplex RT-qPCR was shown to be able to detect individual infections as well as co-infections among the respiratory viruses investigated. It was demonstrated to be a reliable and efficient tool for rapidly and safely diagnosing these infections. Furthermore, this study represents the first report of aMPV strains in Ecuadorian poultry and demonstrates the circulation of aMPV-A, aMPV-B, and GI-13 IBV strains in unvaccinated chicken populations in the country. Thus, it highlights the importance of simultaneously identifying these pathogens in greater detail and on a regular basis in Ecuador.

## 1 Introduction

According to data from 2021 in Ecuador, poultry production represents 2% of the national Gross Domestic Product (GDP), 26% of the total agricultural economy and 77% of agricultural production. It is estimated that annual broiler production in Ecuador is between 230 and 250 million units, which makes it an important sector in the national economy (1). Culturally, given their high protein value, chicken meat and eggs are considered a pillar in the diet of Ecuadorians; therefore, a decrease in production would mean a risk to the social economic balance of the country (1, 2).

Respiratory diseases around the world represent the greatest danger for poultry farms due to high mortality rate and severe economic losses due to delayed growth, decreased egg production, eggshell malformations and infertility, and it may lead to condemnation of a large number of carcasses in slaughterhouses (3). Infectious bronchitis virus (IBV) is one of the most representative causes of respiratory disease in chickens around the world, causing the greatest losses in case of infection by non-vaccine strains (4). IBV is a member of the Gammacoronavirus genus, within the Coronaviridae family and Nidovirales order and has a single-stranded positive RNA genome of approximately 27 kb. The glycoprotein spike (S) is the most immunogenic viral protein and is divided into two subunits: S1 is the main epitope of neutralizing antibodies containing the receptor binding domain (RBD) and S2 containing the transmembrane and C-terminal cholacytoplasmic domains (5). The S1 region is highly variable and is responsible for differentiating strains and ensuring the level of vaccine protection. In 2016, variants based on the S1 region were classified into 32 lineages and 6 genotypes spread worldwide (6). Subsequently, new publications have reported new emerging lineages to be studied (7).

IBV has been circulating in the Americas and Caribbean region for decades, resulting in reports of the emergence of unique IBV strains and variants of the European 793/B and United States Mass genotypes. Notifications have varied by country, but the new strains have been reported several of wild and vaccinated IBV strains circulating in Ecuador, being necessary to use extended detection methods for the virus in order to investigate the impact of the distribution of strains in the country (8).

Avian metapneumovirus (aMPV), which produces upper respiratory tract and reproductive diseases in turkeys, chickens and ducks, is regarded as an important cause of economic and health problems for the poultry industry worldwide (9). aMPV possesses a single-stranded negative RNA genome of approximately 14 kb and belongs to the Metapneumovirus genus within the Pneumoviridae family. Four recognized genotypes of aMPV (A, B, C and D) have been reported according to different genetic characteristics (10). The most widely distributed aMPV lineages are genotypes A and B, reported in virtually all parts of the world as agents of infection in turkeys and chickens (11). The aMPV genotypes C and D have been reported in Europe and Asia and infect mainly ducks and turkeys. However, North American lineages of genotype C have been reported to infect turkeys and chickens causing respiratory syndrome (12). Despite this, it is necessary to consider that different birds can serve as hosts and reservoirs for viral spread (13). Controlled studies have shown that all aMPV genotypes have the ability to infect Galliformes; however, host susceptibility changes according to the viral genotype. Epidemiologically, genotype A is found less and less frequently in poultry due to its low excretion capacity in chickens, while genotype B is increasingly abundant. Circulation of genotype C has been reported in domestic and poultry in the USA and China, and with few reports in France, Italy and South Korea (14, 15); its reported distribution is growing every year (13).

Co-infection of different respiratory viruses have been reported to increase bird mortality (16). Particularly, cases of co-infection between IBV and different genotypes of aMPV have been reported on several occasions (17). IBV infections are thought to weaken the carrier bird and make it more susceptible to subsequent infections that together cause more severe respiratory diseases and increase mortality in poultry (12, 17).

Given the increasing appearance of new genotypes of respiratory viruses in poultry and their constant genetic diversification, it is necessary to perform periodic monitoring to detect the introduction of pathogens not previously reported in the country such as aMPV and the presence of IBV as a causal agent of respiratory disease. This study proposes the development and use of a multiplex RT-qPCR assay for the simultaneous detection of IBV, aMPV-A, aMPV-B and aMPV-C in chickens with respiratory symptoms in Ecuador to determine the possible circulation of these virus in the country.

## 2 Materials and methods

### 2.1 Sample collection and RNA extraction

For this study, 200 tracheal swabs were collected from chickens previously vaccinated with the live-attenuated IBV H120 strain vaccine, but non-vaccinated against other IBV genotypes, neither any genotype of aMPV and showing respiratory symptoms in poultry flocks in three different provinces of Ecuador: Tungurahua, Pichincha y Cotopaxi. The samples were kept at 4°C from their collection until their arrival at the laboratory where they were stored at −20°C until processing. The samples were suspended in 750 μL of 0.1 M PBS pH 7.2. The suspensions were frozen at −80°C for 10 min and heated at 56°C in a water bath for 1 min. Samples were homogenized by vortexing and then centrifuged at 12000 g at 4°C for 30 min and a 250 μL aliquot of the supernatant was taken. Subsequently, the samples were subjected to RNA extraction with TRIZOL Reagent (Thermo Fisher Scientific, Carlsbad, CA, USA) as indicated by the supplier. All procedures conducted in the present study were approved by the Committee on the Care and Use of Laboratory and Domestic Animal resources of the Agency of Regulation and Control of Phytosanitary and Animal Health of Ecuador (AGROCALIDAD), under the number #INT/DA/019.

### 2.2 Reverse transcription reaction

Extracted RNA samples were subjected to reverse transcription (RT) to generate complementary DNA (cDNA) for use in PCR and qPCR assays. For the RT reaction, the SuperScript™ IV Reverse Transcriptase (SS-IV) enzyme was used (Invitrogen, Waltham, MA, USA). First, a denaturation at 65°C for 5 min of 8 μL of extracted RNA with <5 μG of genetic material, 2.5 μM of Random primers, 2.5 μM of Oligo (dT)12 primer, 0.5 mM of each deoxynucleotide triphosphates (dNTP) was performed. After placing on ice for 1 min, a mix of 7 μL containing 1x SS-IV Buffer, 5 mM DTT and 10 U/μL of SS-IV was added. The mix was subjected to 23°C for 10 min then 55°C for 60 min, then 85°C for 10 min and finally cooled to 4°C. The cDNA samples were stored at −20°C until use in assays.

### 2.3 Primer design and standard curve construction

Four primer and probe sets were designed for the simultaneous detection of IBV and aMPV-A, -B and -C (Table 1). All available complete IBV genome sequences and all complete and partial genome sequences for each aMPV genotype were obtained from GenBank and aligned using Clustal Omega with Geneious Prime 2023.0.2 (19). Primers and probes targeting aMPV-A, aMPV-B and aMPV-C were designed manually based on conserved target regions of the viral genomes. To detect IBV, a previously published primer and probe set was modified to consider sequence variations and optimized for multiplexing (18). All primer and probe sequences were analyzed and checked for potential dimer formation using the IDT OligoAnalyzer™ Tool (20). Finally, for each designed sequence, BLAST analysis was performed *in silico* to ensure specificity, and no cross-reactivity was observed.

**Table 1:** Primers and probes used in this study for multiplex qPCR and endpoint PCR.

Virus	Rx	Target	Primer	Sequence	bp	Reference
IBV	qPCR	5’UTR	IBV-F	GCTTTTGAGCCTAGCGTT		Modified from (18)
			IBV-R	GCTTGAAGCCATGTTGTCA		
			IBV-P	CACCACCAGAACCTGTCACCTCAG		
aMPV-A		G gene	aMPVA-F	TAGTCCTATCAGCTTTCGGG		This study
			aMPVA-R	TTGTAGTTTCTGCACTCCTC		
			aMPVA-P	CTGACCTGCACAGTCACTATTGCACTC		
aMPV-B		G gene	aMPVB-F	CTGGAGCAGGAAAGTTTGAG		
			aMPVB-R	TCCTGATTCACAAAGAGCC		
			aMPVB-P	CAAACAATCAACACCACCCAGTACAGC		
aMPV-C		Matrix protein gene	aMPVC-F	CAAGGTGTCCCTTACACTG		
			aMPVC-R	TTGACCTTCAGTATTGGGC		
			aMPVC-P	CAAACCAACACACCTCCTACAGTGCT		
IBV	PCR	S1 gene	XC1+	CACTGGTAATTTTTCAGATGG		(17)
			XC2-	CTCTATAAACACCCTTACA		
aMPV		G gene	G1+	GGGACAAGTATC T/C C/A T/G AT		
			G6-	CTGACAAATTGGTCCTGATT		

A synthetic double-stranded DNA fragment (gBlocks Gene Fragments, IDT) containing the target genomic sequences of all four viruses was used as a positive control for the calibration curve. The DNA concentration in the synthetic DNA fragment was calculated using a NanoDrop 2000 (Thermo Fisher Scientific). Therefore, the Program DNA Copy Number and Dilution Calculator (Thermo Fisher Scientific) was used to determinate the copy number in the stock and the required dilutions to create the standard curve. Nine ten-fold serial dilutions of the DNA standards from 1 to 10^8^ copies were used for the construction of the calibration curves for the multiplex and singleplex assays.

### 2.4 qPCR for virus detection

For standardization of the multiplex and singleplex assays, a 10 μL qPCR reaction was performed using TaqPath™ ProAmp™ Multiplex Master Mix reagent (Applied Biosystems, Waltham, MA, USA), 0.2 μM of each primer, 0.1 μM of each probe, 1 μL of 1:5 diluted cDNA and made up to volume with UltraPure DNase/RNase-Free Distilled Water (Thermo Fisher Scientific). A standard protocol was used as recommended by the manufacturer using the following conditions: 30s at 60°C as pre-read, 5 min at 95°C for initial denaturation; 45 cycles of 10s at 95°C for denaturation, 45 s at 61°C for annealing and 30s at 72°C for extension. The conditions were the same in both multiplex and singleplex assays. The assays were run on the CFX96 Touch Real-Time PCR Detection System (Bio-Rad, Hercules, CA, USA). All samples were run in duplicate, and absolute quantification was performed with the calibration curves. A positive control, ddH2O was used as a negative control and non-template controls were run in each assay to ensure functionality.

### 2.5 Limit of detection and quantification

The limit of detection (LoD) and limit of quantification (LoQ) were determined using the nine serial dilutions of the synthetic DNA control. The LoD was determined by the smallest dilution that could be detected and the LoQ by the smallest dilution that could be quantified in each of the singleplex and multiplex targets.

### 2.6 Specificity of RT-qPCR assay

To determine the specificity of the multiplex assay, an RT-qPCR assay was run using positive controls for common avian viruses available at the UDLA laboratory: Chicken Parvovirus (ChPV), Chicken Astrovirus (CAstV), Avian Nephritis Virus (ANV), Fowl Adenovirus (FAdV) and Newcastle disease virus (NDV). Positive controls were subjected to the same protocol previously described.

### 2.7 Repeatability of the multiplex RT-qPCR assay

To evaluate the intra-assay and inter-assay repeatability and stability of the RT-qPCR assay, five serial dilutions with base 10 of the synthetic double-stranded DNA fragment containing the target genomic sequences of all four viruses were prepared for the assay of RT-qPCR, each of these dilutions was aliquoted separately and stored to −20\u00B0C for -20 °C until use. The mean Ct value and coefficient of variation (CV) were calculated according to the test results, and assay stability was assessed by CV. For inter-assay repeatability, an aliquot of the each of five serial dilutions above described were amplified by RT-qPCR for five times under the same reaction conditions. In each amplification a different aliquot of the five serial dilutions was thawed and used. For intra- assay repeatability, fivefold serially diluted reference samples were prepared, and 5 replicates were performed for each dilution factor.

### 2.8 Endpoint PCR and sanger sequencing

For each virus, positive samples with a number of copies of 1,000 or more were randomly selected and subjected to endpoint PCR with previously published primers (Table 1), with conditions previously described with slight modifications (17). A PCR reaction was formulated with 0.5 μM of each primer, 1x Buffer, 5 mM of each dNTP, 25 mM Mg, 1 U of Platinum Taq DNA polymerase (Thermo Fisher Scientific), 5 μL of 1:5 diluted cDNA and the reaction was completed with UltraPure DNase/RNase-Free Distilled Water (Thermo Fisher Scientific) to 25 μL. Samples were subjected to a PCR protocol using the following amplification conditions: a 5 min cycle at 95°C for initial denaturation; 40 cycles of 30s at 95°C for denaturation, 30s at 57.5°C for primer annealing, and 45 s at 72°C for extension and 10 min at 72°C for final extension. The PCR product was stored at 4°C until confirmation by 2% agarose gel electrophoresis, stained with SYBR® Safe DNA gel stain (Thermo Fisher Scientific) and compared with a TrackIt™ 100 bp DNA Ladder (Thermo Fisher Scientific). Samples with higher intensity bands were subjected to Sanger sequencing. PCR products were purified with ExoSAP-IT™ PCR Product Cleanup Reagent (Applied Biosystems) and the product was sequenced at each primer using BigDye® Terminator v3.1 Cycle Sequencing Kit (Thermo Fisher Scientific) and then read on the 3,500 Series Genetic Analyzer (Applied Biosystems).

### 2.9 Phylogenetic analysis

The obtained sequences were edited using the Geneious software package version 10.2.3 (Biomatters Ltd., Auckland, New Zealand). The obtained consensus sequences of each virus were aligned individually with other IBV or aMPV sequences using the Multiple Sequence Alignment Clustal Omega implemented in Geneious 10.2.3 software package, and the similarities of nucleotides were inferred in the BioEdit Sequence Alignment Editor 7.2.5 software package. The phylogenetic tree for IBV and aMPV was built using a neighbor-joining statistics method, together with a p-distance substitution model and phylogeny test bootstrap model with 1,000 replicates that were integrated in the MEGA version 7 software package (21).

## 3 Results

### 3.1 Standard curves

**Figure 1:**
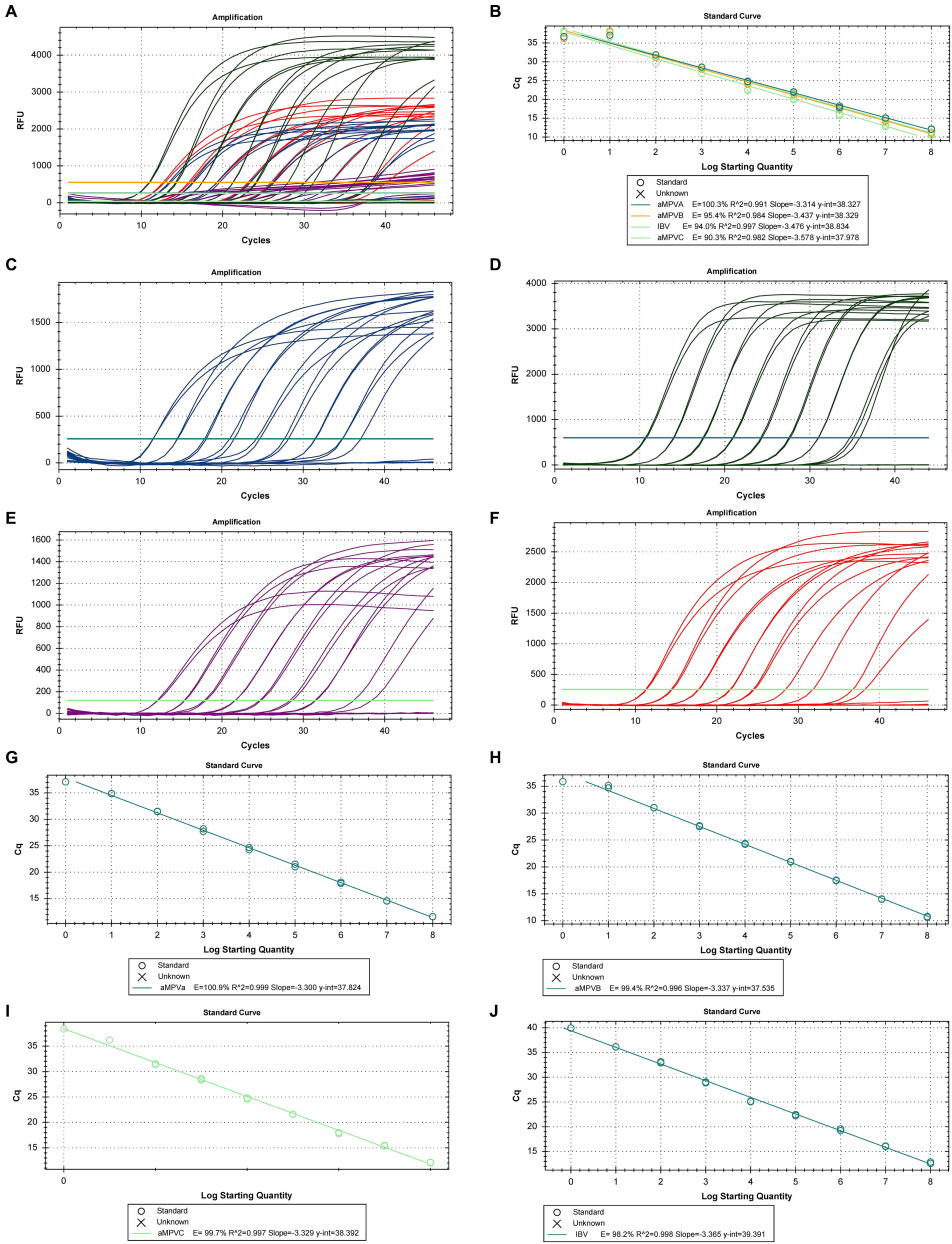
Calibration curves for RT-qPCR for the detection and quantification of aMPV-A, -B, -C, and IBV. **(A)** Amplification Plot of the multiplex calibration curves, **(B)** multiplex calibration curve for the 4 targets, **(C)** amplification Plot of the calibration curve for aMPV-A, **(D)** amplification Plot of the calibration curve for aMPV-B, **(E)** amplification plot of the calibration curve for aMPV-C, **(F)** singleplex calibration curve for aMPV-A, **(G)** singleplex calibration curve for aMPV-B, **(H)** singleplex calibration curve for aMPV-C, **(I)** singleplex calibration curve for IBV, **(J)** singleplex calibration curve for IBV.

The nine ten-fold serial dilutions of the synthetic DNA control were used to generate a multiplex calibration curve for all targets (Figures 1A,B) with efficiencies of 100.3, 95.4, 94.0 and 90.3% for the aMPV-A, aMPV-B, IBV and aMPV-C curves, respectively. In addition, singleplex curves were generated for each of the targets (Figures 1C–F) with efficiencies of 102.6, 99.4 99.7 and 98.2% for aMPV-A, aMPV-B, aMPV-C and IBV curves, respectively (Figures 1G–J).

### 3.2 Run time and limit of detection and quantification

All qPCR were run in standard mode, taking approximately two hours per run. The present assay was able to detect, both in multiplex and singleplex, the DNA control from 10^8^ copies down to 1 copy, thus providing a limit of detection (LoD) and a limit of quantification (LoQ) to 1 copy (Figure 1).

### 3.3 Specificity of RT-qPCR assay

Specificity validation for the multiplex RT-qPCR assay performed with the genetic material of ChPV, CAstV, NDV, ANV, and FAdV showed no amplification for any target, with no nonspecific amplifications, thus this is a specific assay for the simultaneous and specific detection of aMPV-A, B, C and IBV.

### 3.4 Repeatability of assay

Repeatability analysis for multiplex qPCR performed with synthetic double-stranded DNA fragment (g-Block) dilutions showed an inter-assay with a CV of 0.147 to 0.926 as a mean of all targets tested, and intra-assay showed a CV of 0.194 to 1.658 for all targets (Table 2).

**Table 2:** Repeatability assays using g-Block dilutions from 10^8^ to 10^4^ copies of genetic material.

N°C	Inter-assay								Intra-assay							
	aMPV-A	aMPV-B	aMPV-C	IBV	aMPV-A	aMPV-B	aMPV-C	IBV	aMPV-A	aMPV-B	aMPV-C	IBV	aMPV-A	aMPV-B	aMPV-C	IBV
	Cq Mean				Cq St Dev				Cq Mean				Cq St Dev			
10^8^	10.86	11.02	11.09	11.21	0.926	0.160	0.504	0.163	11.26	10.61	11.09	11.25	1.658	0.130	0.847	0.194
10^7^	13.95	14.89	14.83	15.19	0.346	0.172	0.320	0.152	13.43	14.54	14.71	14.99	0.425	0.449	0.397	0.420
10^6^	16.93	17.79	17.74	18.10	0.404	0.242	0.420	0.203	16.27	17.47	18.03	17.87	0.327	0.383	0.452	0.289
10^5^	20.77	21.74	21.72	21.19	0.267	0.224	0.240	0.147	21.68	20.79	21.83	21.76	0.204	0.514	0.853	0.477
10^4^	23.71	24.42	24.45	24.86	0.539	0.249	0.590	0.248	23.96	24.06	24.56	24.58	0.435	0.222	0.295	0.206

### 3.5 Multiplex RT-qPCR for virus detection

**Figure 2:**
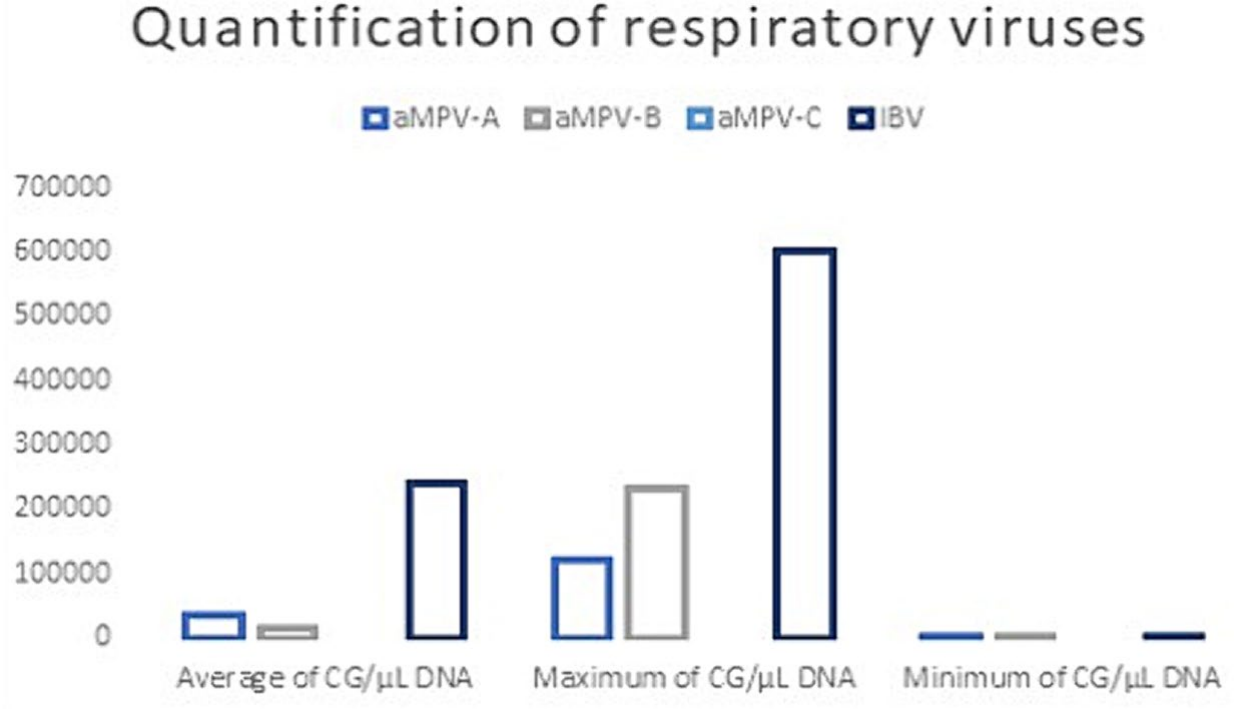
Quantification of positive samples with the detection method used for aMPV-A, aMPV-B, aMPV-C, and IBV. Average, maximum and minimum.

Of the 200 samples analyzed, 114 (57%) were positive for IBV, 25 (12.5%) were positive for aMPV-B, 13 (6.5%) were positive for aMPV-A, and 61 (30.5%) were negative for all four viruses. No positive samples for aMPV-C were found (Tables 3, 4). IBV-positive samples showed the highest viral load compared to aMPV-A and aMPV-B, showing up to 21-fold higher gene copies (Figure 2). None of the runs showed unspecific products in either the samples, non-template controls or the negative controls.

**Table 3:** Summary of positive samples and detection for respiratory viruses aMPV-A, aMPV-B, aMPV-C, and IBV in tracheal samples using multiplex qPCR.

Detecction of respiratory viruses					
Target	Number of total of samples	Number of positives samples	Average of CG/μL DNA	Maximum of CG/μL DNA	Minimum of CG/μL DNA
aMPV-A	200	13	34,534	123,123	121
aMPV-B		25	13,123	233,900	1,230
aMPV-C		0	0	0	0
IBV		114	741,264	9,200,344	1

CG, Gene Copies.

**Table 4:** Number of positive samples for IBV and aMPV alone and in coinfection with each other with different combinations.

Combinations			N° (+)
IBV			101
	aMPV-A		3
		aMPV-B	13
IBV	aMPV-A		3
IBV		aMPV-B	6
	aMPV-A	aMPV-B	2
IBV	aMPV-A	aMPV-B	4
Negative samples			61
Total samples			200

N° (+), Number of positive samples.

### 3.6 Coinfection of IBV and aMPV

Multiplex qPCR assays on the 200 tracheal samples showed coinfection between IBV and aMPV-A and B. Of the 15 co-infection positive samples, 4 samples were found to be positive for IBV, aMPV-A and aMPV-B genomes. The most common combination was aMPV-B and IBV with 6 samples in coinfection and the least common was aMPV-A and aMPV-B with 2 samples in coinfection (Table 4).

### 3.7 Phylogenetic analysis

#### 3.7.1 IBV

**Figure 3:**
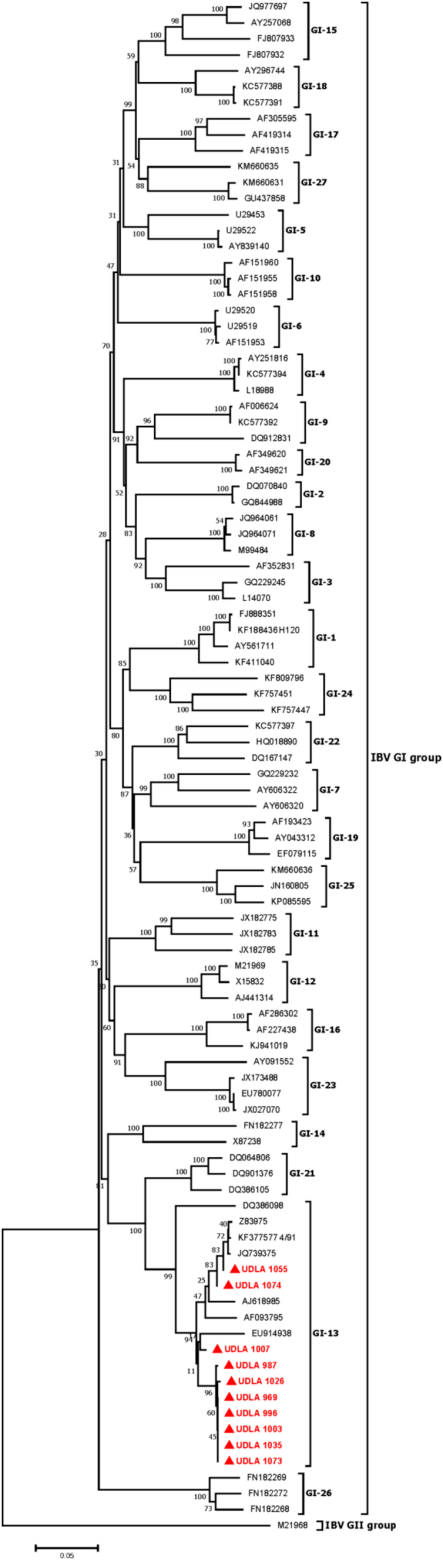
Phylogenetic three for IBV. Sequences were aligned using the CLUSTAL W method in ClustalX2 2.1. The phylogenetic tree was constructed using MEGA 7 Software package. Numbers along the branches refer to bootstrap values for 1,000 replicates. The scale bar represents the number of substitutions per site. In red and market with ▲ are all sequences obtained in this study.

The phylogenetic analysis showed IBV strains belonging to GI-13 group, where one sample was clustered with the vaccine strain 4/91 (KF377577) and the others was grouped separately (Figure 3). The sequences obtained in the present study showed 95.63 to 100% nucleotide (NT) similarity between them. The sequences here obtained were then compared with other IBV sequences from GI-13 IBV group including a 4/91 vaccine sequence, where two samples (UDLA 1055 and UDLA 1074) have 99.56 to 100% NT similarity with the vaccine sequence, whereas the rest of the isolated sequences showed a 95.63–98.47% NT similarity compared to the 4/91 sequence (Supplementary material).

#### 3.7.2 aMPV

**Figure 4:**
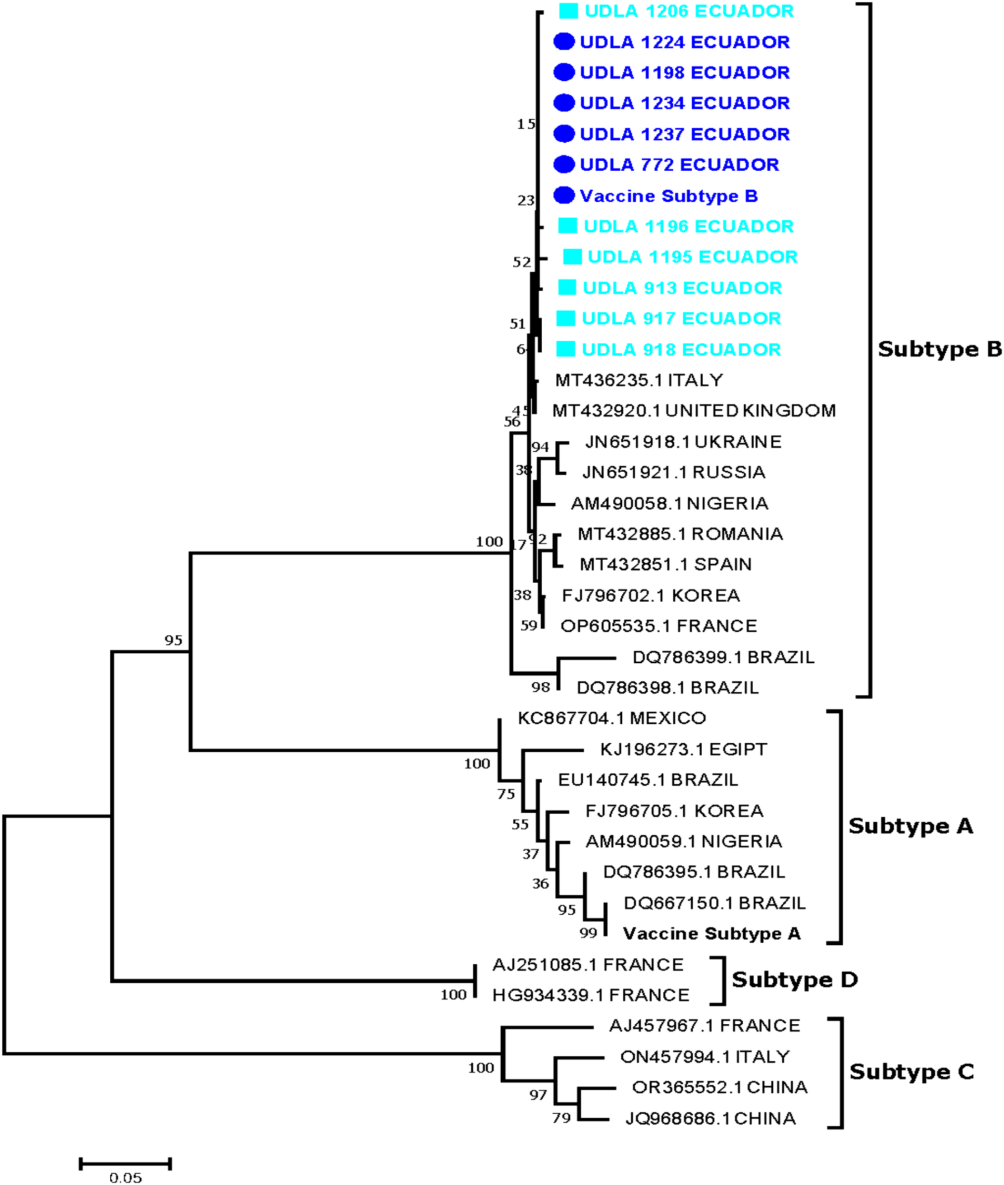
Sequences were aligned using the CLUSTAL W method in ClustalX2 2.1. The phylogenetic tree was constructed using MEGA 7 Software package. Numbers along the branches refer to bootstrap values for 1,000 replicates. The scale bar represents the number of substitutions per site. In blue and market with ■ are sequences obtained in this study classified as vaccine-derived strains; In sky blue and market with ● are sequences obtained in this study classified as vaccine strains.

Ten positive samples for aMPV-B were successfully amplified and Sanger-sequenced, while none of the 13 samples that were positive for aMPV-A with the RT-qPCR assay could be amplified by end-point PCR. The phylogenetic analysis confirmed that the aMPV strains belonged to genotype B phylogenetic group, where all samples were clustered in a unique group including the vaccine aMPV-B (Figure 4). The sequences obtained in the present study showed a 99.55 to 100% nucleotide (NT) similarity between them. Five of the isolated aMPV-B sequences (UDLA 772, UDLA 1224, UDLA 1234, UDLA 1237, UDLA 1198) showed 100% NT similarity with the aMPV-B vaccine strain and thus classified as vaccine strains, whereas and the other five Ecuadorian aMPV-B sequences had 99.55–99.77% NT similarity compared to vaccine aMPV-B sequence and thus classified as vaccine-derived strains (Supplementary material).

## 4 Discussion

Respiratory viruses like IBV and aMPV pose a threat to poultry, Therefore, it is imperative to conduct diagnostic procedures, such as the one described in this study, when symptoms arise (20). Prior research has performed the concurrent identification of aMPV genotypes, while other studies have developed techniques for detecting genotypes A and B of aMPV together with IBV. However, this study presents, for the first time, a method capable of simultaneously detecting genotypes A, B, and C of aMPV and IBV (4, 11, 13, 14, 22), showing that the assay has greater detection coverage of these respiratory pathogens, promoting epidemiological surveillance, and lowering diagnostic costs. It has been previously detailed that the use of hydrolysis probe-based qPCR is more sensitive, rapid and suitable for the detection of viral pathogens (22, 23). Multiplex qPCR allows simultaneous detection of related pathogens that are of clinical interest in coinfection. The new multiplex RT-qPCR assay using hydrolysis probes developed and standardized in this study for simultaneous detection of IBV and aMPV-A, B, and C shows a LoD and LoQ of down to 1 copy, an efficiency between 90 and 100% in multiplex assay, and a repeatability with a CV of 0.363 for inter-assay and 0.459 for intra-assay in multiplex. The quality criteria of these reactions are crucial for the advancement of multiplex RT-qPCR. The vast quantity of primers and probes employed in this technique can result in possible non-specific amplification, thus requiring the usage of meticulously designed oligonucleotides (24, 25).

The present study shows the emergence of aMPV strains in solitary and co-infection with IBV circulating in chickens with respiratory disease in Ecuador. Since no aMPV infection had been previously described in Ecuador, this indicates the virus could be considered as a new cause of respiratory viral diseases in poultry that entails the emergence of vaccine-derived strains (26). Of the 200 samples tested, 57% were positive for IBV, which is acknowledged as responsible for the third largest economic losses in poultry worldwide, causing respiratory, renal and reproductive disease as well as deformation of the eggshell and damage to the egg white contents (27). Furthermore, aMPV can lead to a reduction in egg production and compromised eggshell integrity (26, 27). Consequently, the simultaneous presence of IBV and aMPV infections poses a significant financial threat to poultry farmers (3, 4, 17, 28). Frequent occurrences of simultaneous infections of these two viruses have been well-documented in North America, Europe, and Asia. Therefore, it is necessary to adopt detection techniques that can identify both IBV and the most prevalent strains of aMPV at the same time (13–15). While aMPV-C was not found in the sample, it is important to regularly monitor for this genotype due to the growing number of reports of its presence in chickens. In addition, given the low number of samples used, a more extensive study is necessary to estimate the presence of the genotype in the country. In the phylogenetic analysis performed, IBV sequences corresponding to GI-13 group reported in Morocco for the first time were identified (6, 28). Of the 10 sequenced strains, two sequences were identified as vaccinal and 8 sequences as wild, considering that it has been demonstrated that the S1 of the vaccinal strain 4/91 and the wild strains 793/B (GI-13) differ by 0.6% (28, 29). The presence of wild IBV strains in Ecuador poses a health risk for poultry, provided that predominant vaccination strain used in Ecuador is GI-1 IBV group (Massachusetts). It should be noted that in Ecuador the presence of possible Qx strains (GI-19) has been reported (8); however, as in this study, only a fragment of S1 was used. Therefore, it is necessary to sequence the complete sequence of S1 in order to precisely ascertain the source of these wild strains that are responsible for causing infectious bronchitis (6, 30, 31). On the other hand, in the analysis of aMPV genotype B, 5 sequences identical to the vaccine and 6 sequences with a similarity of up to 99.56% were found. These 6 sequences are cataloged as “vaccine-derived strains” because the differences are up to 2 nucleotides in the genome (17, 26). Vaccines for genotypes A and B of aMPV have been circulating in Ecuador since the late 1970s and no wild-type strains for either genotypes of aMPV have been previously reported (12, 23, 32, 33). The existence of these vaccine-derived strains poses an increasing threat, as there have been sporadic observations of the reversion of the vaccine virulence in birds exhibiting respiratory symptoms. This reversion enables the strains to regain their ability to cause infection, leading to the sickness and mortality associated with it (30, 32, 33). Unvaccinated chickens are exhibiting symptoms of aMPV infection, which could be caused by vaccine-derived strains that have regained their ability to cause disease (21, 27). Nevertheless, further research is required to ascertain the underlying cause. Hence, it is recommended to utilize this new multiplex RT-qPCR assay to accelerate the surveillance of this virus in conjunction with IBV.

## 5 Conclusion

In conclusion, the use of molecular methods such as multiplex RT-qPCR assays are a viable alternative for the monitoring of pathogens such as viruses, as they work efficiently and with great sensitivity. The method developed for the simultaneous detection of avian respiratory viruses IBV, aMPV-A, aMPV-B and aMPV-C showed high efficiency and effectiveness for continuous use. Regular monitoring of viral infections impacting poultry production is necessary. To ensure accurate and safe long-term control of their spread, it is advisable to employ multiplex approaches as a reference.

## Data availability statement

The original contributions presented in the study are included in the article/Supplementary material, further inquiries can be directed to the corresponding author.

## Ethics statement

The animal study was approved by Committee on the Care and Use of Laboratory and Domestic Animal resources of the Agency of Regulation and Control of Phytosanitary and Animal Health of Ecuador (AGROCALIDAD), under the number #INT/DA/019. The study was conducted in accordance with the local legislation and institutional requirements.

## Author contributions

AL-G: Data curation, Formal analysis, Investigation, Writing – original draft. CM: Methodology, Writing – review & editing. SS-P: Writing – review & editing, Investigation, Methodology. DC: Writing – review & editing, Methodology. DT: Writing – review & editing. HA: Writing – review & editing. LN: Conceptualization, Funding acquisition, Investigation, Methodology, Project administration, Supervision, Writing – review & editing.
