# Supplementary material for: Simultaneous detection of infectious bronchitis virus and avian metapneumovirus genotypes A, B, and C by multiplex RT-qPCR assay in chicken tracheal samples in Ecuador
Source: Front Vet Sci. 2024 Jul 18;11:1387172. doi: 10.3389/fvets.2024.1387172 (PMC11292056; doi:10.3389/fvets.2024.1387172)
Supplement: Supplementary file 1 [file Data_Sheet_1.PDF]

Comparison of the nucleotide of Ecuadorian samples of IBV with other sequences reported of this virus

	1.	2.	3.	4.	5.	6.	7.	8.	9.	10.	11.	12.	13.	14.	15.	16.	17.	18.	19.	20.	21.
1. KF188436.1 H120 vac		94.13%	97.64%	99.94%	83.23%	77.73%	77.73%	78.82%	77.51%	77.51%	77.51%	77.51%	77.51%	77.51%	77.29%	77.02%	76.70%	76.89%	76.89%	77.51%	77.91%
2. KF411040 GI-1	94.13%		95.16%	94.29%	78.06%	79.69%	79.69%	80.35%	79.04%	79.04%	79.04%	79.04%	79.04%	79.04%	78.82%	76.46%	76.95%	76.65%	76.52%	77.33%	77.79%
3. AY561711 GI-1	97.64%	95.16%		97.58%	77.81%	78.82%	78.82%	79.91%	78.60%	78.60%	78.60%	78.60%	78.60%	78.60%	78.38%	77.19%	76.76%	77.07%	77.07%	77.87%	77.93%
4. FJ888351 GI-1	99.94%	94.29%	97.58%		77.51%	77.51%	77.51%	78.60%	77.29%	77.29%	77.29%	77.29%	77.29%	77.29%	77.07%	76.96%	76.46%	76.83%	76.83%	77.45%	77.63%
5. KF377577 4/91 vac	83.23%	78.06%	77.81%	77.51%		100%	99.56%	98.47%	96.07%	95.85%	95.85%	95.85%	95.85%	95.85%	95.63%	92.65%	92.50%	95.49%	96.41%	99.63%	99.76%
6. UDLA 1055 GI-13	77.73%	79.69%	78.82%	77.51%	100%		99.56%	98.47%	96.07%	95.85%	95.85%	95.85%	95.85%	95.85%	95.63%	88.43%	95.85%	96.29%	97.60%	100%	100%
7. UDLA 1074 GI-13	77.73%	79.69%	78.82%	77.51%	99.56%	99.56%		98.47%	96.07%	95.85%	95.85%	95.85%	95.85%	95.85%	95.63%	88.43%	95.41%	95.85%	97.16%	99.56%	99.56%
8. UDLA 1007 GI-13	78.82%	80.35%	79.91%	78.60%	98.47%	98.47%	98.47%		97.60%	97.38%	97.38%	97.38%	97.38%	97.38%	97.16%	89.96%	96.07%	96.07%	97.38%	98.47%	98.47%
9. UDLA 987 GI-13	77.51%	79.04%	78.60%	77.29%	96.07%	96.07%	96.07%	97.60%		99.78%	99.78%	99.78%	99.78%	99.78%	99.56%	90.39%	94.98%	94.32%	96.51%	96.07%	96.07%
10. UDLA 1073 GI-13	77.51%	79.04%	78.60%	77.29%	95.85%	95.85%	95.85%	97.38%	99.78%		100%	100%	100%	100%	99.78%	90.61%	95.20%	94.10%	96.29%	95.85%	95.85%
11. UDLA 969 GI-13	77.51%	79.04%	78.60%	77.29%	95.85%	95.85%	95.85%	97.38%	99.78%	100%		100%	100%	100%	99.78%	90.61%	95.20%	94.10%	96.29%	95.85%	95.85%
12. UDLA 996 GI-13	77.51%	79.04%	78.60%	77.29%	95.85%	95.85%	95.85%	97.38%	99.78%	100%	100%		100%	100%	99.78%	90.61%	95.20%	94.10%	96.29%	95.85%	95.85%
13. UDLA 1003 GI-13	77.51%	79.04%	78.60%	77.29%	95.85%	95.85%	95.85%	97.38%	99.78%	100%	100%	100%		100%	99.78%	90.61%	95.20%	94.10%	96.29%	95.85%	95.85%
14. UDLA 1035 GI-13	77.51%	79.04%	78.60%	77.29%	95.85%	95.85%	95.85%	97.38%	99.78%	100%	100%	100%	100%		99.78%	90.61%	95.20%	94.10%	96.29%	95.85%	95.85%
15. UDLA 1026 GI-13	77.29%	78.82%	78.38%	77.07%	95.63%	95.63%	95.63%	97.16%	99.56%	99.78%	99.78%	99.78%	99.78%	99.78%		90.39%	94.98%	93.89%	96.07%	95.63%	95.63%
16. DQ386098 GI-13	77.02%	76.46%	77.19%	76.96%	92.65%	88.43%	88.43%	89.96%	90.39%	90.61%	90.61%	90.61%	90.61%	90.61%	90.39%		89.88%	92.40%	93.26%	92.58%	92.58%
17. EU914938 GI-13	76.70%	76.95%	76.76%	76.46%	92.50%	95.85%	95.41%	96.07%	94.98%	95.20%	95.20%	95.20%	95.20%	95.20%	94.98%	89.88%		91.73%	92.96%	92.16%	92.44%
18. AF093795 GI-13	76.89%	76.65%	77.07%	76.83%	95.49%	96.29%	95.85%	96.07%	94.32%	94.10%	94.10%	94.10%	94.10%	94.10%	93.89%	92.40%	91.73%		95.67%	95.36%	95.49%
19. AJ618985 GI-13	76.89%	76.52%	77.07%	76.83%	96.41%	97.60%	97.16%	97.38%	96.51%	96.29%	96.29%	96.29%	96.29%	96.29%	96.07%	93.26%	92.96%	95.67%		96.54%	96.35%
20. Z83975 GI-13	77.51%	77.33%	77.87%	77.45%	99.63%	100%	99.56%	98.47%	96.07%	95.85%	95.85%	95.85%	95.85%	95.85%	95.63%	92.58%	92.16%	95.36%	96.54%		99.51%
21. JQ739375 GI-13	77.91%	77.79%	77.93%	77.63%	99.76%	100%	99.56%	98.47%	96.07%	95.85%	95.85%	95.85%	95.85%	95.85%	95.63%	92.58%	92.44%	95.49%	96.35%	99.51%	

Comparison of the nucleotide of Ecuadorian samples of aMPV-B with other sequences reported of this virus, and aMPV-B vaccine and aMPV-A vaccine.

	1.	2.	3.	4.	5.	6.	7.	8.	9.	10.	11.	12.
1. Vaccine Subtype B		100%	100%	100%	100%	100%	99.77%	99.77%	99.77%	99.55%	99.77%	99.77%
2. UDLA 772 ECU	100%		100%	100%	100%	100%	99.77%	99.77%	99.77%	99.55%	99.77%	99.77%
3. UDLA 1224 ECU	100%	100%		100%	100%	100%	99.77%	99.77%	99.77%	99.55%	99.77%	99.77%
4. UDLA 1234 ECU	100%	100%	100%		100%	100%	99.77%	99.77%	99.77%	99.55%	99.77%	99.77%
5. UDLA 1237 ECU	100%	100%	100%	100%		100%	99.77%	99.77%	99.77%	99.55%	99.77%	99.77%
6. UDLA 1198 ECU	100%	100%	100%	100%	100%		99.77%	99.77%	99.77%	99.55%	99.77%	99.77%
7. UDLA 913 ECU	99.77%	99.77%	99.77%	99.77%	99.77%	99.77%		99.55%	99.55%	99.32%	99.55%	99.55%
8. UDLA 917 ECU	99.77%	99.77%	99.77%	99.77%	99.77%	99.77%	99.55%		100%	99.32%	99.55%	99.55%
9. UDLA 918 ECU	99.77%	99.77%	99.77%	99.77%	99.77%	99.77%	99.55%	100%		99.32%	99.55%	99.55%
10. UDLA 1195 ECU	99.55%	99.55%	99.55%	99.55%	99.55%	99.55%	99.32%	99.32%	99.32%		99.32%	99.32%
11. UDLA 1196 ECU	99.77%	99.77%	99.77%	99.77%	99.77%	99.77%	99.55%	99.55%	99.55%	99.32%		99.55%
12. UDLA 1206 ECU	99.77%	99.77%	99.77%	99.77%	99.77%	99.77%	99.55%	99.55%	99.55%	99.32%	99.55%	
13. DQ786399.1 BRAZIL	92.21%	92.21%	92.21%	92.21%	92.21%	92.21%	92.21%	92.21%	92.21%	91.88%	91.88%	91.88%
14. DQ786398.1 BRAZIL	95.96%	95.96%	95.96%	95.96%	95.96%	95.96%	95.96%	95.96%	95.96%	95.65%	95.65%	95.65%
15. OP605535.1 FRANCE	98.78%	98.78%	98.78%	98.78%	98.78%	98.78%	98.78%	98.78%	98.78%	98.17%	98.47%	98.47%
16. MT436235.1 ITALY	99.51%	99.51%	99.51%	99.51%	99.51%	99.51%	99.27%	99.27%	99.27%	99.03%	99.27%	99.27%
17. FJ796702.1 KOREA	98.62%	98.62%	98.62%	98.62%	98.62%	98.62%	98.62%	98.62%	98.62%	98.28%	98.62%	98.28%
18. AM490058.1 NIGERIA	98.32%	98.32%	98.32%	98.32%	98.32%	98.32%	98.04%	98.04%	98.04%	97.77%	98.04%	98.04%
19. MT432885.1 ROM	97.76%	97.76%	97.76%	97.76%	97.76%	97.76%	97.76%	97.76%	97.76%	97.48%	97.48%	97.48%
20. JN651921.1 RUSSIA	97.44%	97.44%	97.44%	97.44%	97.44%	97.44%	97.20%	97.20%	97.20%	97.44%	97.20%	97.20%
21. MT432851.1 SPAIN	97.72%	97.72%	97.72%	97.72%	97.72%	97.72%	97.72%	97.72%	97.72%	97.15%	97.44%	97.44%
22. JN651918.1 UKRAINE	97.44%	97.44%	97.44%	97.44%	97.44%	97.44%	97.20%	97.20%	97.20%	96.97%	97.20%	97.20%
23. MT432920.1 UK	99.67%	99.67%	99.67%	99.67%	99.67%	99.67%	99.67%	99.67%	99.67%	99.34%	99.67%	99.34%
24. Vaccine Subtype A	56.11%	56.11%	56.11%	56.11%	56.11%	56.11%	56.11%	56.11%	56.11%	56.11%	56.11%	56.11%
25. DQ667150.1 BRAZIL	56.11%	56.11%	56.11%	56.11%	56.11%	56.11%	56.11%	56.11%	56.11%	56.11%	56.11%	56.11%
26. EU140745.1 BRAZIL	60.34%	60.34%	60.34%	60.34%	60.34%	60.34%	60.34%	60.34%	60.34%	60.34%	60.34%	60.34%
27. DQ786395.1 BRAZIL	58.53%	58.53%	58.53%	58.53%	58.53%	58.53%	58.53%	58.53%	58.53%	58.53%	58.53%	58.53%
28. KJ196273.1 EGIPT	58.15%	58.15%	58.15%	58.15%	58.15%	58.15%	57.91%	58.15%	58.15%	57.91%	58.15%	58.39%
29. FJ796705.1 KOREA	59.38%	59.38%	59.38%	59.38%	59.38%	59.38%	59.13%	59.38%	59.38%	59.13%	59.38%	59.64%
30. KC867704.1 MEXICO	64.60%	64.60%	64.60%	64.60%	64.60%	64.60%	64.60%	64.60%	64.60%	64.60%	64.60%	64.60%
31. AM490059.1 NIGERIA	59.12%	59.12%	59.12%	59.12%	59.12%	59.12%	58.89%	59.12%	59.12%	58.89%	59.12%	59.36%
32. JQ968686.1 CHINA	18.14%	18.14%	18.14%	18.14%	18.14%	18.14%	18.14%	18.14%	18.14%	17.93%	18.35%	17.93%
33. OR365552.1 CHINA	16.31%	16.31%	16.31%	16.31%	16.31%	16.31%	16.15%	16.46%	16.46%	16.15%	16.46%	16.15%
34. AJ457967.1 FRANCE	17.51%	17.51%	17.51%	17.51%	17.51%	17.51%	17.36%	17.67%	17.67%	17.36%	17.67%	17.36%
35. ON457994.1 ITALY	18.57%	18.57%	18.57%	18.57%	18.57%	18.57%	18.57%	18.57%	18.57%	18.35%	18.78%	18.35%
36. AJ251085.1 FRANCE	55.63%	55.63%	55.63%	55.63%	55.63%	55.63%	55.41%	55.41%	55.41%	55.18%	55.41%	55.63%
37. HG934339.1 FRANCE	55.63%	55.63%	55.63%	55.63%	55.63%	55.63%	55.41%	55.41%	55.41%	55.18%	55.41%	55.63%

	13.	14.	15.	16.	17.	18.	19.	20.	21.	22.	23.	24.
1. Vaccine Subtype B	92.21%	95.96%	98.78%	99.51%	98.62%	98.32%	97.76%	97.44%	97.72%	97.44%	99.67%	56.11%
2. UDLA 772 ECU	92.21%	95.96%	98.78%	99.51%	98.62%	98.32%	97.76%	97.44%	97.72%	97.44%	99.67%	56.11%
3. UDLA 1224 ECU	92.21%	95.96%	98.78%	99.51%	98.62%	98.32%	97.76%	97.44%	97.72%	97.44%	99.67%	56.11%
4. UDLA 1234 ECU	92.21%	95.96%	98.78%	99.51%	98.62%	98.32%	97.76%	97.44%	97.72%	97.44%	99.67%	56.11%
5. UDLA 1237 ECU	92.21%	95.96%	98.78%	99.51%	98.62%	98.32%	97.76%	97.44%	97.72%	97.44%	99.67%	56.11%
6. UDLA 1198 ECU	92.21%	95.96%	98.78%	99.51%	98.62%	98.32%	97.76%	97.44%	97.72%	97.44%	99.67%	56.11%
7. UDLA 913 ECU	92.21%	95.96%	98.78%	99.27%	98.62%	98.04%	97.76%	97.20%	97.72%	97.20%	99.67%	56.11%
8. UDLA 917 ECU	92.21%	95.96%	98.78%	99.27%	98.62%	98.04%	97.76%	97.20%	97.72%	97.20%	99.67%	56.11%
9. UDLA 918 ECU	92.21%	95.96%	98.78%	99.27%	98.62%	98.04%	97.76%	97.20%	97.72%	97.20%	99.67%	56.11%
10. UDLA 1195 ECU	91.88%	95.65%	98.17%	99.03%	98.28%	97.77%	97.48%	97.44%	97.15%	96.97%	99.34%	56.11%
11. UDLA 1196 ECU	91.88%	95.65%	98.47%	99.27%	98.62%	98.04%	97.48%	97.20%	97.44%	97.20%	99.67%	56.11%
12. UDLA 1206 ECU	91.88%	95.65%	98.47%	99.27%	98.28%	98.04%	97.48%	97.20%	97.44%	97.20%	99.34%	56.11%
13. DQ786399.1 BRAZIL		97.06%	92.86%	92.53%	95.52%	91.81%	92.16%	91.56%	91.56%	90.91%	95.83%	55.83%
14. DQ786398.1 BRAZIL	97.06%		96.58%	96.27%	96.21%	95.70%	95.34%	95.34%	95.34%	94.72%	96.04%	54.30%
15. OP605535.1 FRANCE	92.86%	96.58%		99.08%	100%	98.94%	98.77%	97.86%	98.78%	97.55%	99.01%	57.01%
16. MT436235.1 ITALY	92.53%	96.27%	99.08%		98.97%	98.47%	97.76%	97.32%	97.72%	97.32%	100%	56.56%
17. FJ796702.1 KOREA	95.52%	96.21%	100%	98.97%		98.88%	98.62%	97.93%	98.62%	97.24%	98.94%	57.50%
18. AM490058.1 NIGERIA	91.81%	95.70%	98.94%	98.47%	98.88%		97.49%	97.68%	97.57%	97.68%	98.47%	55.31%
19. MT432885.1 ROM	92.16%	95.34%	98.77%	97.76%	98.62%	97.49%		97.20%	99.42%	96.64%	97.69%	55.66%
20. JN651921.1 RUSSIA	91.56%	95.34%	97.86%	97.32%	97.93%	97.68%	97.20%		96.87%	99.07%	97.69%	56.11%
21. MT432851.1 SPAIN	91.56%	95.34%	98.78%	97.72%	98.62%	97.57%	99.42%	96.87%		96.58%	97.69%	56.11%
22. JN651918.1 UKRAINE	90.91%	94.72%	97.55%	97.32%	97.24%	97.68%	96.64%	99.07%	96.58%		97.03%	56.11%
23. MT432920.1 UK	95.83%	96.04%	99.01%	100%	98.94%	98.47%	97.69%	97.69%	97.69%	97.03%		56.56%
24. Vaccine Subtype A	55.83%	54.30%	57.01%	56.56%	57.50%	55.31%	55.66%	56.11%	56.11%	56.11%	56.56%	
25. DQ667150.1 BRAZIL	55.83%	54.30%	57.01%	56.56%	57.50%	55.31%	55.66%	56.11%	56.11%	56.11%	56.56%	100%
26. EU140745.1 BRAZIL	56.48%	58.62%	61.21%	60.78%	58.10%	55.03%	59.91%	60.34%	60.34%	60.34%	61.04%	95.41%
27. DQ786395.1 BRAZIL	56.48%	56.68%	59.45%	58.99%	58.10%	55.03%	58.06%	58.53%	58.53%	58.53%	58.99%	100%
28. KJ196273.1 EGIPT	53.90%	56.21%	58.72%	57.76%	56.55%	52.46%	59.13%	57.91%	58.69%	58.15%	59.74%	91.28%
29. FJ796705.1 KOREA	54.22%	55.90%	58.41%	59.64%	56.90%	51.80%	60.78%	59.64%	58.69%	59.64%	59.74%	94.04%
30. KC867704.1 MEXICO	57.29%	59.62%	62.86%	65.04%	59.14%	56.97%	64.16%	64.60%	64.60%	64.60%	62.32%	95.59%
31. AM490059.1 NIGERIA	53.90%	55.90%	58.41%	57.71%	56.55%	53.91%	59.09%	57.86%	58.43%	58.11%	59.74%	95.87%
32. JQ968686.1 CHINA	24.35%	23.60%	22.94%	18.14%	24.48%	20.51%	21.57%	17.72%	21.65%	17.93%	22.77%	18.81%
33. OR365552.1 CHINA	24.35%	23.60%	22.94%	15.40%	24.48%	17.73%	21.57%	16.06%	21.65%	16.06%	22.77%	18.81%
34. AJ457967.1 FRANCE	24.03%	23.29%	22.63%	16.34%	24.14%	18.58%	22.13%	16.98%	21.37%	16.98%	22.44%	17.89%
35. ON457994.1 ITALY	24.03%	23.29%	22.63%	18.57%	24.14%	20.26%	22.13%	18.14%	21.37%	18.35%	22.44%	18.35%
36. AJ251085.1 FRANCE	53.57%	54.66%	54.43%	54.74%	54.14%	50.56%	56.30%	55.24%	54.70%	55.01%	55.12%	56.42%
37. HG934339.1 FRANCE	53.57%	54.66%	54.43%	54.74%	54.14%	50.56%	56.30%	55.24%	54.70%	55.01%	55.12%	56.42%

	25.	26.	27.	28.	29.	30.	31.	32.	33.	34.	35.	36.	37.
1. Vaccine Subtype B	56.11%	60.34%	58.53%	58.15%	59.38%	64.60%	59.12%	18.14%	16.31%	17.51%	18.57%	55.63%	55.63%
2. UDLA 772 ECU	56.11%	60.34%	58.53%	58.15%	59.38%	64.60%	59.12%	18.14%	16.31%	17.51%	18.57%	55.63%	55.63%
3. UDLA 1224 ECU	56.11%	60.34%	58.53%	58.15%	59.38%	64.60%	59.12%	18.14%	16.31%	17.51%	18.57%	55.63%	55.63%
4. UDLA 1234 ECU	56.11%	60.34%	58.53%	58.15%	59.38%	64.60%	59.12%	18.14%	16.31%	17.51%	18.57%	55.63%	55.63%
5. UDLA 1237 ECU	56.11%	60.34%	58.53%	58.15%	59.38%	64.60%	59.12%	18.14%	16.31%	17.51%	18.57%	55.63%	55.63%
6. UDLA 1198 ECU	56.11%	60.34%	58.53%	58.15%	59.38%	64.60%	59.12%	18.14%	16.31%	17.51%	18.57%	55.63%	55.63%
7. UDLA 913 ECU	56.11%	60.34%	58.53%	57.91%	59.13%	64.60%	58.89%	18.14%	16.15%	17.36%	18.57%	55.41%	55.41%
8. UDLA 917 ECU	56.11%	60.34%	58.53%	58.15%	59.38%	64.60%	59.12%	18.14%	16.46%	17.67%	18.57%	55.41%	55.41%
9. UDLA 918 ECU	56.11%	60.34%	58.53%	58.15%	59.38%	64.60%	59.12%	18.14%	16.46%	17.67%	18.57%	55.41%	55.41%
10. UDLA 1195 ECU	56.11%	60.34%	58.53%	57.91%	59.13%	64.60%	58.89%	17.93%	16.15%	17.36%	18.35%	55.18%	55.18%
11. UDLA 1196 ECU	56.11%	60.34%	58.53%	58.15%	59.38%	64.60%	59.12%	18.35%	16.46%	17.67%	18.78%	55.41%	55.41%
12. UDLA 1206 ECU	56.11%	60.34%	58.53%	58.39%	59.64%	64.60%	59.36%	17.93%	16.15%	17.36%	18.35%	55.63%	55.63%
13. DQ786399.1 BRAZIL	55.83%	56.48%	56.48%	53.90%	54.22%	57.29%	53.90%	24.35%	24.35%	24.03%	24.03%	53.57%	53.57%
14. DQ786398.1 BRAZIL	54.30%	58.62%	56.68%	56.21%	55.90%	59.62%	55.90%	23.60%	23.60%	23.29%	23.29%	54.66%	54.66%
15. OP605535.1 FRANCE	57.01%	61.21%	59.45%	58.72%	58.41%	62.86%	58.41%	22.94%	22.94%	22.63%	22.63%	54.43%	54.43%
16. MT436235.1 ITALY	56.56%	60.78%	58.99%	57.76%	59.64%	65.04%	57.71%	18.14%	15.40%	16.34%	18.57%	54.74%	54.74%
17. FJ796702.1 KOREA	57.50%	58.10%	58.10%	56.55%	56.90%	59.14%	56.55%	24.48%	24.48%	24.14%	24.14%	54.14%	54.14%
18. AM490058.1 NIGERIA	55.31%	55.03%	55.03%	52.46%	51.80%	56.97%	53.91%	20.51%	17.73%	18.58%	20.26%	50.56%	50.56%
19. MT432885.1 ROM	55.66%	59.91%	58.06%	59.13%	60.78%	64.16%	59.09%	21.57%	21.57%	22.13%	22.13%	56.30%	56.30%
20. JN651921.1 RUSSIA	56.11%	60.34%	58.53%	57.91%	59.64%	64.60%	57.86%	17.72%	16.06%	16.98%	18.14%	55.24%	55.24%
21. MT432851.1 SPAIN	56.11%	60.34%	58.53%	58.69%	58.69%	64.60%	58.43%	21.65%	21.65%	21.37%	21.37%	54.70%	54.70%
22. JN651918.1 UKRAINE	56.11%	60.34%	58.53%	58.15%	59.64%	64.60%	58.11%	17.93%	16.06%	16.98%	18.35%	55.01%	55.01%
23. MT432920.1 UK	56.56%	61.04%	58.99%	59.74%	59.74%	62.32%	59.74%	22.77%	22.77%	22.44%	22.44%	55.12%	55.12%
24. Vaccine Subtype A	100%	95.41%	100%	91.28%	94.04%	95.59%	95.87%	18.81%	18.81%	17.89%	18.35%	56.42%	56.42%
25. DQ667150.1 BRAZIL		95.41%	100%	91.28%	94.04%	95.59%	95.87%	18.81%	18.81%	17.89%	18.35%	56.42%	56.42%
26. EU140745.1 BRAZIL	95.41%		99.53%	95.20%	97.38%	99.51%	99.56%	18.34%	18.78%	17.90%	18.78%	58.95%	58.95%
27. DQ786395.1 BRAZIL	100%	99.53%		95.33%	98.13%	100%	100%	20.09%	19.63%	18.69%	19.63%	58.88%	58.88%
28. KJ196273.1 EGIPT	91.28%	95.20%	95.33%		94.29%	95.52%	96.41%	18.10%	16.29%	16.75%	17.66%	55.39%	55.39%
29. FJ796705.1 KOREA	94.04%	97.38%	98.13%	94.29%		97.76%	98.49%	18.26%	14.70%	15.01%	18.47%	56.48%	56.48%
30. KC867704.1 MEXICO	95.59%	99.51%	100%	95.52%	97.76%		99.78%	16.14%	16.14%	14.80%	15.25%	61.43%	61.43%
31. AM490059.1 NIGERIA	95.87%	99.56%	100%	96.41%	98.49%	99.78%		17.82%	16.64%	16.77%	17.37%	55.97%	55.97%
32. JQ968686.1 CHINA	18.81%	18.34%	20.09%	18.10%	18.26%	16.14%	17.82%		96.41%	89.52%	95.51%	19.53%	19.53%
33. OR365552.1 CHINA	18.81%	18.78%	19.63%	16.29%	14.70%	16.14%	16.64%	96.41%		84.90%	93.11%	18.70%	18.70%
34. AJ457967.1 FRANCE	17.89%	17.90%	18.69%	16.75%	15.01%	14.80%	16.77%	89.52%	84.90%		91.02%	19.14%	19.14%
35. ON457994.1 ITALY	18.35%	18.78%	19.63%	17.66%	18.47%	15.25%	17.37%	95.51%	93.11%	91.02%		20.38%	20.38%
36. AJ251085.1 FRANCE	56.42%	58.95%	58.88%	55.39%	56.48%	61.43%	55.97%	19.53%	18.70%	19.14%	20.38%		100%
37. HG934339.1 FRANCE	56.42%	58.95%	58.88%	55.39%	56.48%	61.43%	55.97%	19.53%	18.70%	19.14%	20.38%	100%	
